# Concurrent double aortic arch and circumflex aorta repair in a symptomatic child: a case report

**DOI:** 10.1186/s13019-022-01907-6

**Published:** 2022-06-07

**Authors:** Christopher G. Hurtado, Jennifer S. Nelson

**Affiliations:** 1 Department of Cardiovascular Services, Nemours Children’s Hospital, 6535 Nemours Parkway, Orlando, FL 32827 USA; 2 Department of Surgery, University of Central Florida College of Medicine, 6850 Lake Nona Blvd, Orlando, FL 32827 USA

**Keywords:** Double aortic arch, Circumflex aorta, Surgery technique, Case report

## Abstract

Double aortic arch with circumflex aorta is a rare type of vascular ring. Symptoms result from external compression of the trachea and esophagus. The best surgical approach for patients with double arch and circumflex aorta is debated, and options include the highly complex aortic uncrossing procedure. Herein we describe the surgical treatment of a patient with concurrent double aortic arch and circumflex aorta by division of the non-dominant arch and ligamentum arteriosum, plication and posterior tacking of the diverticulum of Kommerell. This left thoracotomy approach provided complete symptom resolution.

## Background

Double aortic arch is the most common type of vascular ring anomaly accounting for 30–50% of all cases [1]. Double aortic arches may be further categorized based on arch dominance, and a dominant right arch with smaller left arch is most common. A circumflex aorta is defined by retroesophageal crossing of the aorta to the contralateral side superior to the level of the tracheal carina. In rare cases, a double aortic arch and circumflex aorta may be seen in the same patient. The most commonly reported symptoms of vascular rings include stridor (57%), recurrent upper respiratory tract infections (27%), cough (21%), dysphagia (15%) and respiratory distress (10%) [2]. In double aortic arch, symptoms typically develop in early infancy, however, dysphagia may not present until the introduction of solid foods.

The best surgical approach for patients with a double aortic arch and concurrent circumflex aorta is debated because prior reports suggest that division of the non-dominant aortic arch and ligamentum arteriosum via thoracotomy may not adequately relieve tracheal and esophageal compression [3, 4]. Drs Planche and LaCoeur-Gayet performed the first aortic uncrossing procedure in three patients with circumflex aorta who had persistent symptoms despite initial surgery. Aortic uncrossing is a complex operation generally utilizing cardiopulmonary bypass ± deep hypothermic circulatory arrest that relocates the aortic arch anterior to the trachea and esophagus. Modifications may allow for the avoidance of cardiopulmonary bypass, however, aortic uncrossing remains a more complex procedure with a higher risk of major complication such as bilateral recurrent laryngeal nerve injury, compared to a thoracotomy approach for division of the lesser arch and ligamentum arteriosum [5, 6]. It is unclear whether primary presenting symptomatology (e.g. dysphagia vs. noisy breathing vs. both) may indicate which patients are most likely to have persistent or recurrent symptoms following vascular ring division via thoracotomy. We present the case of a child with severe dysphagia secondary to a double aortic arch and circumflex aorta and discuss a straight-forward surgical treatment option.

## Case presentation

A 9-year-old boy with a past medical history of autism and seizures was evaluated for an 8-year history of dysphagia to solid foods. The patient indicated that solid food would get “stuck” so he habitually chewed for prolonged periods of time before spitting it out. His diet consisted mostly of soup and nutritional supplements. The patients’ parents denied any history of stridor or other respiratory symptoms.

**Fig. 1:**
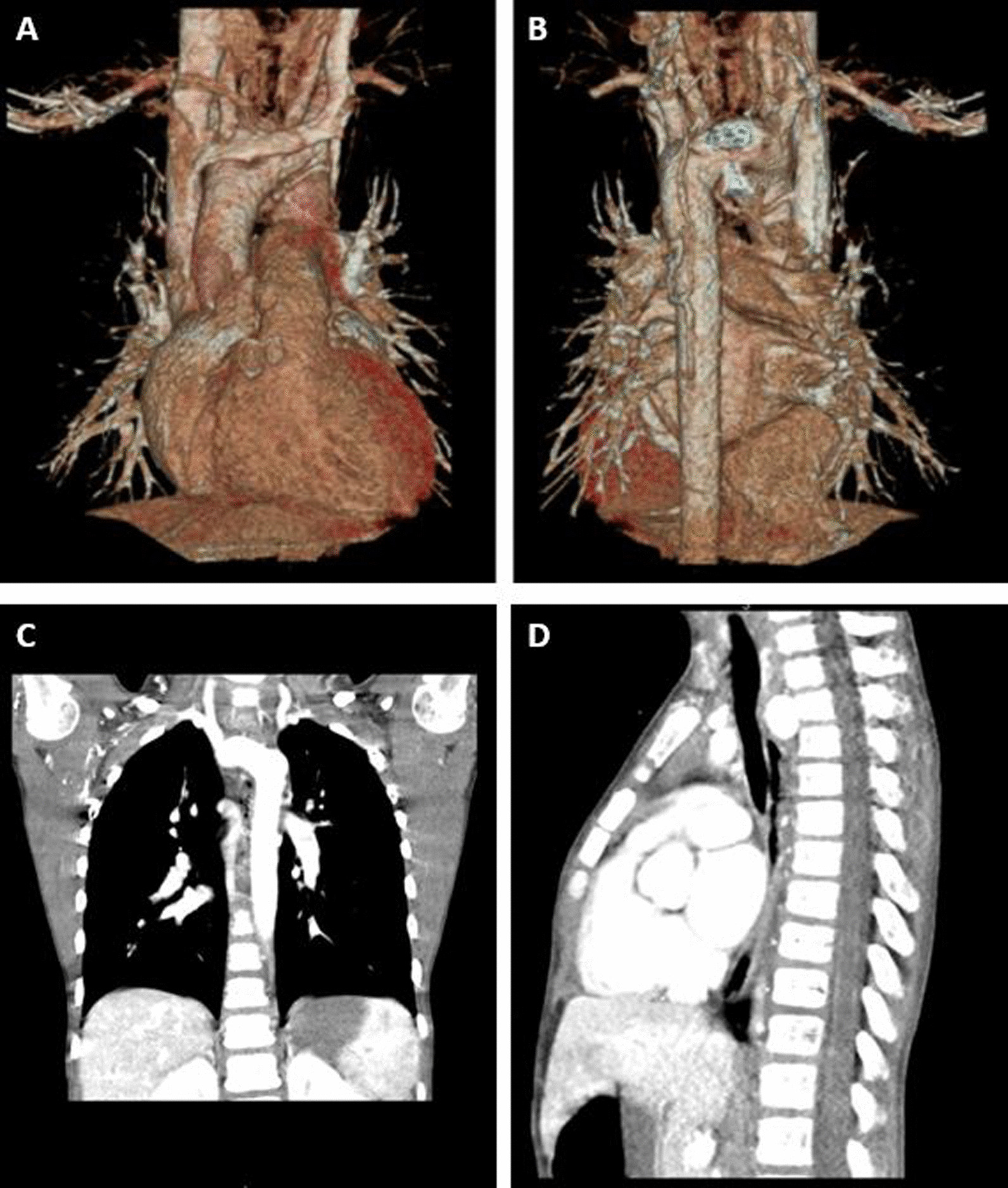
Preoperative imaging. Three-dimensional computed tomographic reconstruction showing **A** anterior view of double aortic arch, and **B** posterior view of circumflex aorta. **C** Coronal and **D** Sagittal computed tomographic images of circumflex aorta crossing right to left posterior to the trachea and superior to the carina

On initial exam, the patient was 25th percentile in weight and 17th percentile for height with a BMI of 16. His diagnostic workup included a CT angiogram of the chest and an echocardiogram that revealed a double aortic arch (right dominant with smaller left arch and prominent Kommerell’s diverticulum). The patient also had a circumflex aorta with left sided descending thoracic aorta (Fig. 1A–B). The vascular ring resulted in significant compression of the esophagus and mild right-sided and posterior compression of the trachea (Fig. 1C–D). Due to the presence of a complex vascular ring and severe dysphagia, the patient was referred for surgery.

## Surgical technique

**Fig. 2:**
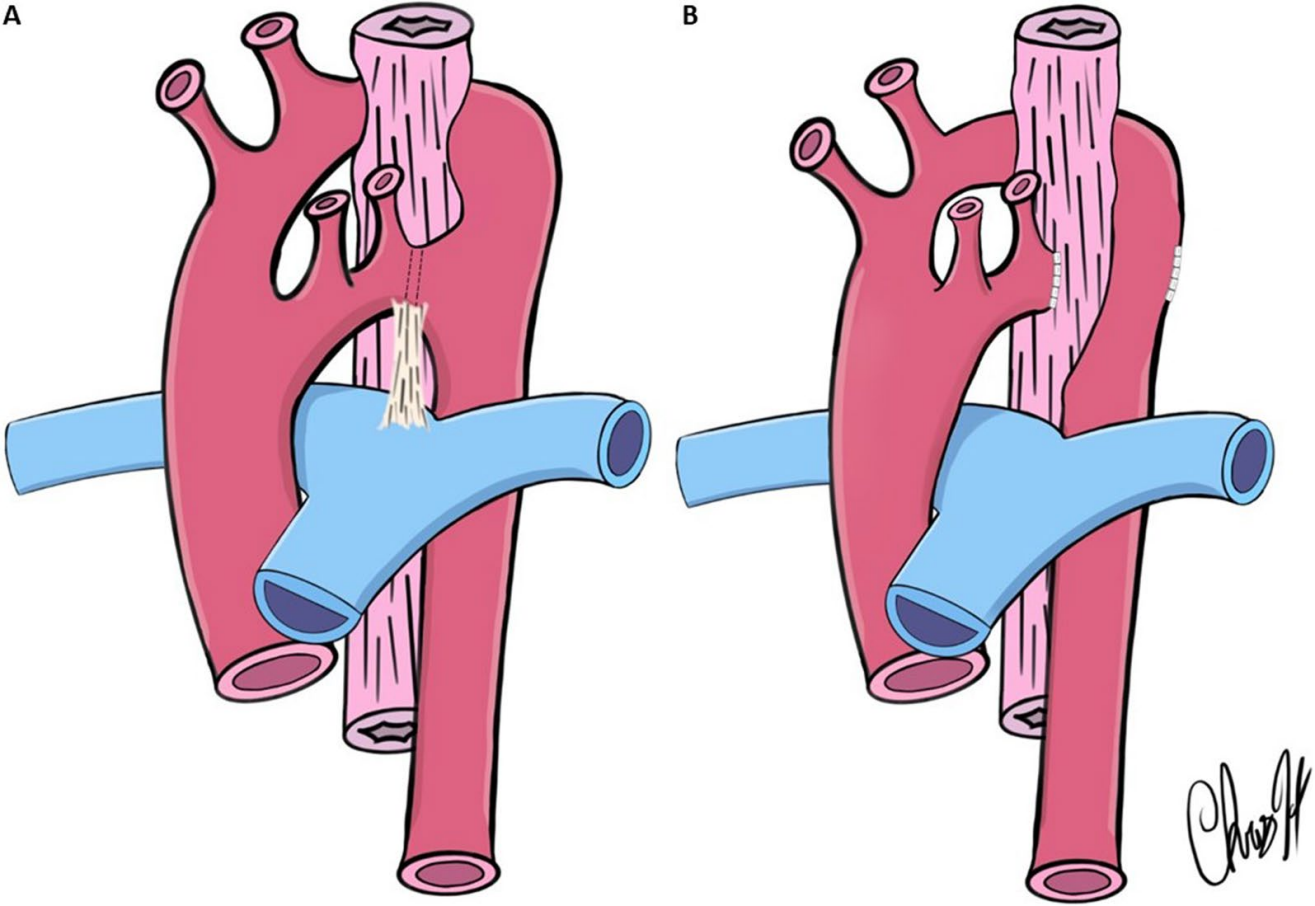
Cartoon drawing of preoperative anatomy **A** and postoperative anatomy **B**. The ligamentum arteriosum and the non-dominant left arch were divided, and the Kommerell’s Diverticulum was plicated and tacked posteriorly, thus providing relief of lateral esophageal compression from the complete vascular ring

A left posterolateral thoracotomy was performed, utilizing the third intercoastal space. The left subclavian artery, ligamentum arteriosum, left recurrent laryngeal nerve, and left aortic arch were identified. The left subclavian artery, Kommerell’s diverticulum, ligamentum arteriosum, and left arch were dissected out. The ligamentum arteriosum was suture ligated and divided. Vascular control was obtained for division of the left arch using a vascular clamp placed distal to the takeoff of the left subclavian artery on the smaller left arch, and another clamp on Kommerell’s diverticulum. The left aortic arch was divided, and the ends were oversewn which also effectively plicated the Kommerell’s diverticulum (Fig. 2). There was significant relief of compression of the esophagus after division of the left arch. Adventitial bands crossing the esophagus were divided. The diverticulum of Kommerell was tacked posteriorly to the prevertebral fascia/parietal pleura to provide further relief of esophageal compression. There were no blood pressure gradients observed, nor residual esophageal compression appreciated at the end of the operation.

The patient received an erector spinae block at the completion of the procedure and was extubated in the operating room. There were no postoperative complications. The chest tube was removed on the first postoperative day. The patient tolerated a regular diet including solids on the second postoperative day, and he was discharged home the following day. At 1-year follow-up he remains symptom free and is eating meats and other solids without dysphagia.

## Discussion and conclusions

In this patient with severe dysphagia and circumflex aorta with retroesophageal dominant right aortic arch and left-sided descending thoracic aorta, division of the non-dominant left arch and ligamentum arteriosum and plication and tacking the diverticulum of Kommerell posteriorly provided complete symptom relief in a straight-forward approach, without incurring the risks of an aortic uncrossing procedure. The limitations of this report are common to other case reports and include the lack of a comparison group and follow-up limited to one year with the inability to predict recurrence risk. For greater generalizability, future studies are needed to assess the effectiveness of this surgical technique in patients with similar anatomic considerations.

Both the aortic uncrossing procedure and its described variations are associated with significant risk of major complications. Kamran et al. [6] described eight patients undergoing aortic uncrossing procedures due to circumflex aorta; five had concurrent double aortic arch and two patients suffered postoperative bilateral vocal cord paralysis. Russel et al. [7] performed an aortic uncrossing procedure in four patients with circumflex aorta. One patient suffered respiratory insufficiency requiring reintubation. Another patient required postoperative temporary tracheostomy and suffered right-sided Horner syndrome and bilateral recurrent laryngeal nerve paresis.

In the surgical treatment of other vascular rings, some have argued for the resection of Kommerell’s Diverticulum to prevent symptom recurrence [2, 8]. For example, of 300 patients with either double aortic arch or right aortic arch with left ligamentum, Backer and colleagues noted 18 patients presented with residual or persistent symptoms following ligamentum division alone. They recommend resecting ‘significant’ Kommerell’s diverticula (defined as > 1.5 times the size of the distal subclavian artery) to prevent aneurysm formation, dissection or inadequate compression relief, and symptoms recurrence [2].

**Fig. 3:**
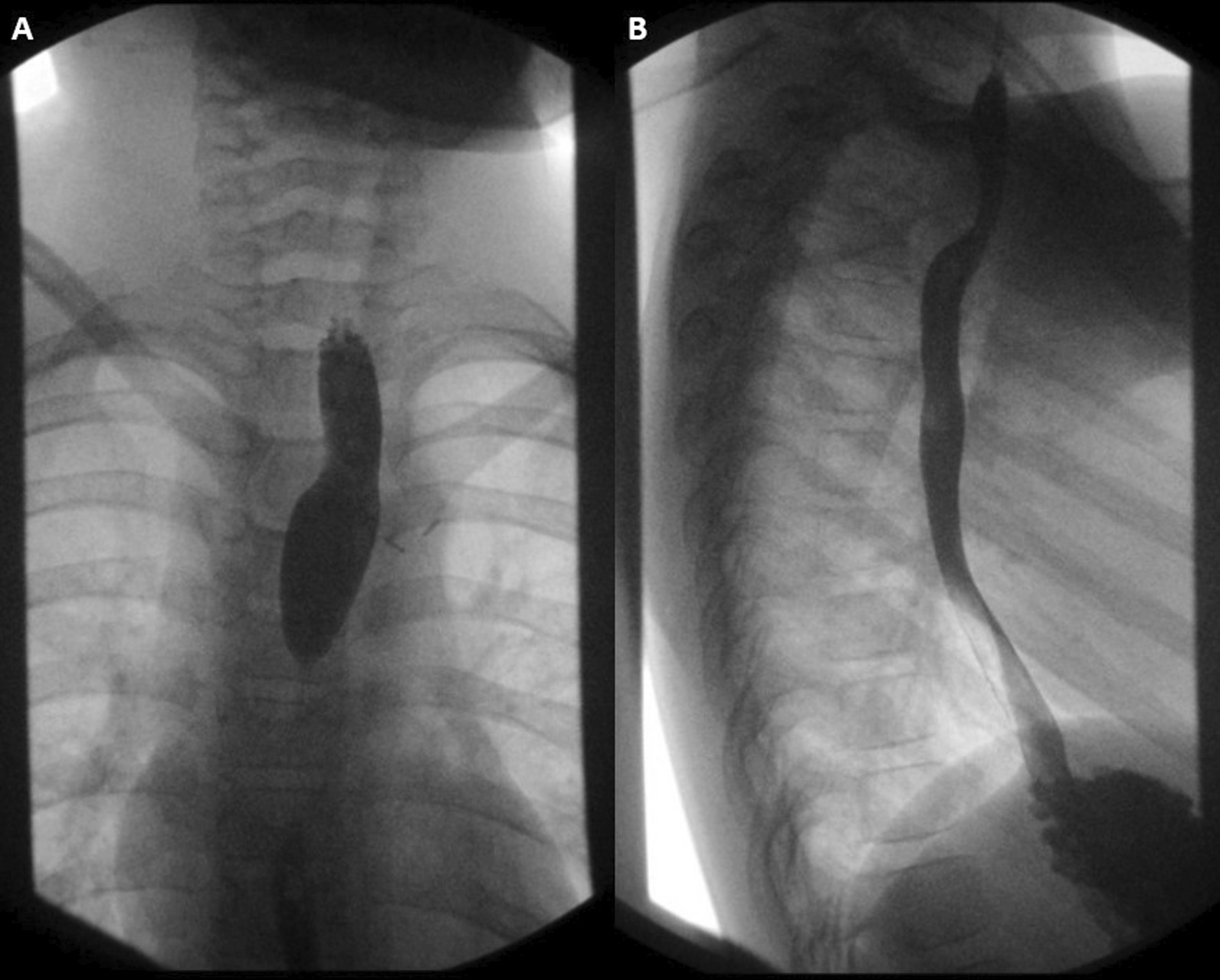
Post-operative imaging including anterior/posterior **A** and lateral **B** views of single contrast upper GI study utilizing oral administration of barium. Here, the dominant right arch is left in its native position posterior to the esophagus, and, as anticipated, posterior indentation of the esophagus is seen, but with compensatory left lateral extrusion of the esophagus made possible by division of the vascular ring

In our patient, the Kommerell’s diverticulum was significant (original size 15 × 14 mm) and was plicated to reduce its size prior to posterior tacking. As this operation leaves the dominant right arch in its original position posterior to the aorta, follow-up esophagram shows the expected posterior indentation at this level, but compensatory left lateral extrusion of the esophagus and normal caliber (Fig. 3). In this and other vascular rings with Kommerell’s diverticula, we have not seen recurrence at mid-term follow-up after ring division with this approach. If patients treated with this technique develop recurrent symptoms later in life, aortic uncrossing may still be performed via first-time sternotomy. Extra-anatomic bypass has also been described as a definitive treatment in adults [9]. In select children with double aortic arch and circumflex aorta, aortic uncrossing is unnecessary. Here, a straight-forward approach to double arch and ligamentum division was safe and effective at relieving dysphagia.

## Data Availability

Data sharing is not applicable to this article as no datasets were generated or analyzed during the current study.

## References

[1] Yoshimura N, Fukahara K, Yamashita A (2020). Congenital vascular ring. Surg Today.

[2] Backer CL, Mongé MC, Popescu AR, Eltayeb OM, Rastatter JC, Rigsby CK (2016). Vascular rings. Semin Pediatr Surg.

[3] Planché C, Lacour-Gayet F (1984). Décroisement aortique pour aorte circonflexe compressive. Trois observations [aortic uncrossing for compressive circumflex aorta. 3 cases]. Presse Med.

[4] Backer CL, Mongé MC, Russell HM, Popescu AR, Rastatter JC, Costello JM (2014). Reoperation after vascular ring repair. Semin Thorac Cardiovasc Surg Pediatr Card Surg Annu.

[5] Subramaniam KG, Marimuthu K, Manohar K, Verma S, Cherian KM (2011). Anterior arch translocation for coarctation of circumflex aorta using median sternotomy without cardiopulmonary bypass. J Thorac Cardiovasc Surg.

[6] Kamran A, Friedman KG, Jennings RW, Baird CW (2020). Aortic uncrossing and tracheobronchopexy corrects tracheal compression and tracheobronchomalacia associated with circumflex aortic arch. J Thorac Cardiovasc Surg.

[7] Russell HM, Rastatter JC, Backer CL (2013). The aortic uncrossing procedure for circumflex aorta. Oper Tech Thorac Cardiovasc Surg.

[8] Luciano D, Mitchell J, Fraisse A, Lepidi H, Kreitmann B, Ovaert C (2015). Kommerell diverticulum should be removed in children with vascular ring and aberrant left subclavian artery. Ann Thorac Surg.

[9] Garg P, Bishnoi AK, Sharma P (2013). Ventral aorta repair for retroesophageal circumflex aortic arch in an adult. J Card Surg.

